# Effect of Experimental Cutaneous Hand Pain on Corticospinal Excitability and Short Afferent Inhibition

**DOI:** 10.3390/brainsci6040045

**Published:** 2016-09-29

**Authors:** Catherine Mercier, Martin Gagné, Karen T. Reilly, Laurent J. Bouyer

**Affiliations:** 1 Center for Interdisciplinary Research in Rehabilitation and Social Integration, Québec, QC G1M 2S8, Canada; catherine.mercier@rea.ulaval.ca (C.M.); martin.gagne.77@gmail.com (M.G.); 2 Department of Rehabilitation, Laval University, Québec, QC G1V 0A6, Canada; 3 ImpAct Team, Lyon Neuroscience Research Center, INSERM U1028, CNRS UMR5292, Bron 69500, France; karen.reilly@inserm.fr; 4 University Claude Bernard Lyon 1, Lyon F-69000, France

**Keywords:** sensorimotor integration, nociception, transcranial magnetic stimulation, motor cortex, ulnar nerve

## Abstract

Sensorimotor integration is altered in people with chronic pain. While there is substantial evidence that pain interferes with neural activity in primary sensory and motor cortices, much less is known about its impact on integrative sensorimotor processes. Here, the short latency afferent inhibition (SAI) paradigm was used to assess sensorimotor integration in the presence and absence of experimental cutaneous heat pain applied to the hand. Ulnar nerve stimulation was combined with transcranial magnetic stimulation to condition motor evoked potentials (MEPs) in the first dorsal interosseous muscle. Four interstimulus intervals (ISI) were tested, based on the latency of the N20 component of the afferent sensory volley (N20−5 ms, N20+2 ms, N20+4 ms, N20+10 ms). In the PAIN condition, MEPs were smaller compared to the NEUTRAL condition (*p* = 0.005), and were modulated as a function of the ISI (*p* = 0.012). Post-hoc planned comparisons revealed that MEPs at N20+2 and N20+4 were inhibited compared to unconditioned MEPs. However, the level of inhibition (SAI) was similar in the PAIN and NEUTRAL conditions. This suggests that the interplay between pain and sensorimotor integration is not mediated through direct and rapid pathways as assessed by SAI, but rather might involve higher-order integrative areas.

## 1. Introduction

Moving our limbs and interacting within the world involves the generation of a motor intention and motor commands, monitored by feedback arising from proprioceptive, tactile and visual information. This constant integration of sensorimotor information is essential to maintain a coherent representation of our body as well as to adapt to new conditions. When movement and sensation are discordant, however, sensorimotor integration can break down. For example, after amputation, phantom hand movements lead to activity in muscles that do not normally control the hand [1,2,3], which results in a discordance between the afferent feedback and the motor command. In a seminal paper, Harris proposed that this type of discordance could result in the sensation of pain and might be related to some pathological pain states such as phantom limb pain [4].

Support for Harris’ hypothesis comes from research with chronic pain patients showing that artificially creating sensorimotor disturbances can increase pain [5,6] and that artificially restoring sensorimotor congruence can sometimes decrease pain [7,8,9]. While Harris’ hypothesis has raised a lot of interest from a clinical perspective, the neural mechanisms underlying discordance” or “sensorimotor congruence” remain elusive. The extensive body of research on sensorimotor reorganisation in various chronic pain populations has focused mainly on map and excitability changes within the sensory or motor cortex [10,11,12,13,14], with very few studies specifically targeting the interaction between the sensory and motor systems. One reason for this might be because it is a complex phenomenon that is technically challenging to assess.

Sensory inputs influence motor cortex activity through several pathways of varying complexity, including direct monosynaptic connections from the sensory to motor cortex [15,16]. Relatively direct pathways between primary sensory and motor regions can be probed using transcranial magnetic stimulation with a protocol called short afferent inhibition (SAI) [17]. In this protocol, brief electrical stimulation of a peripheral nerve is paired with a single transcranial magnetic stimulation (TMS) pulse over the primary motor cortex. When the TMS pulse is delivered, a few milliseconds after the afferent volley arrives at the primary sensory cortex the muscle response evoked by the TMS is inhibited.

So far, SAI has been assessed in populations with chronic pain in only two studies. One of these found that patients with chronic shoulder pain (*n* = 8) had less SAI than control subjects in the M1 representation of the infraspinatus muscle, an important dynamic stabiliser of the shoulder [18]. Interestingly, SAI was normalised (i.e., increased toward control subject levels) 30 min after a suprascapular nerve block, but returned to pre-block levels one week later. The other study examined SAI in patients with complex regional pain syndrome (CRPS) type 1 and found that most patients (six out of eight) exhibited significant SAI in the M1 representation of a hand muscle [19], but since they made no direct quantitative comparison between the amount of SAI in patients and controls it is unclear whether SAI in CRPS patients is fully normal. As underlined by Turton and colleagues [19], the very nature of CRPS and other chronic pain conditions sometimes makes it very difficult for patients to tolerate even non-nociceptive sensory stimuli. This, together with the large inter-subject variability in SAI observed in healthy subjects [20] (meaning that a large sample would be needed to show differences between patients and controls) makes it difficult to investigate the effect of pain on SAI using patient populations.

A complementary approach to studying the effect of pain on SAI is to test healthy subjects using an experimental pain model. Although experimental pain models have inherent limitations, acute pain in healthy volunteers reproduces some features of chronic pain such as interfering with S1 and M1 activity [21,22,23,24,25,26,27,28,29,30,31] and interacting with sensorimotor integration [32]. Another advantage of experimental pain models is that they are compatible with within-subject designs, which reduces the problem of inter-subject variability in pain responses and SAI. To date, only one study has assessed SAI and pain using a pre-post, within-subject design. This study examined SAI in the M1 representation of the first dorsal interosseous (FDI) muscle before, during, and after hypertonic saline infusion into the same muscle [28]. The results showed that corticomotor output was reduced both during pain and after its resolution, and that although SAI had a tendency to decrease at all time points, the only significant reduction in SAI occurred immediately after pain resolution. While studying sensorimotor integration in a muscle pain model is clearly relevant in view of the evidence that muscle pain interferes with the processing of signals from non-nociceptive muscle afferents [30,31], the most severe sensorimotor disturbances in chronic pain patients are typically reported in clinical populations that have other types of pain, such as CRPS patients.

The aim of the present study was to assess the effect of hand pain that does not arise from the muscle (cutaneous heat pain) on SAI in the M1 representation of FDI. One big advantage of the heat pain model (compared, for example, with the hypertonic saline or the capsaicin model) is that it permits the application of brief nociceptive stimuli, and is therefore compatible with a within-subject, single-session experiment in which SAI can be repeatedly assessed in both the presence and absence of pain.

## 2. Materials and Methods

### 2.1. Participants

Ten healthy subjects (six females) with an average age of 25.8 years (SD 4.1) participated in the study. All subjects completed a medical questionnaire and inclusion criteria included absence of any pain, neurological disorders, musculoskeletal disorders or contraindications for TMS. The study was approved by the local ethics committee at the Institut de Réadaptation en Déficience Physique de Québec (CER #2011-258) and all participants provided written informed consent.

### 2.2. General Procedure

**Figure 1:**
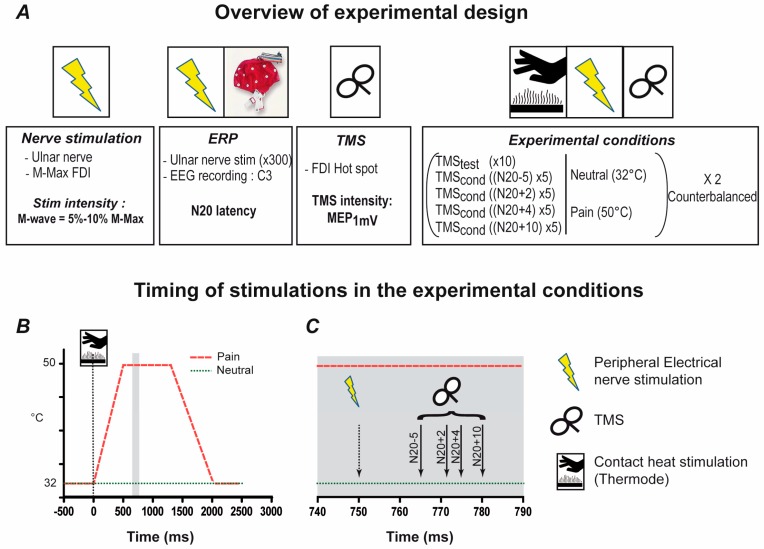
Experimental protocol. (**A**) The experimental design and the timeline of the experiment showing the three steps in the Preparation Phase plus the Experimental Phase; (**B**) The timing of TMS relative to the Heat Pain stimulus (TMS_test_ trials); (**C**) The timing of the TMS relative to the electrical stimulation (TMS_cond_ trials). Note that the shaded area shown in (**C**) is a magnification of the shaded area in (**B**).

Each subject took part in a single experimental session that consisted of a Preparation Phase and an Experimental Phase (see Figure 1 for a schematic representation of the experimental protocol). In the Preparation Phase the stimulation parameters (intensity and timing) to be used in the experiment were established. In the Experimental Phase SAI was tested under a condition that produced no pain (NEUTRAL) or with a thermal stimulation that produced transient heat pain (PAIN). Throughout the session subjects were comfortably seated with their right arm resting on a support, their elbow flexed at approximately 90 degrees and their hand in neutral position.

#### 2.2.1. Preparation Phase

The first step in the preparation phase was to set the intensity of the peripheral electrical stimulus. Bipolar surface electrodes were placed just proximal to the pisiform bone, separated by one centimeter with the cathode proximal to the anode and ulnar nerve stimulation was achieved using a Digitimer DS7A unit (Digitimer Limited, Letchworth Garden City, UK) delivering 200 μs square pulses. Stimulation intensity was gradually increased to obtain the M-Max in the FDI muscle and then gradually decreased to reach the intensity that produced an M-wave of between 5% and 10% of the M-max. This intensity was then used throughout the experimental session.

The second step in the preparation phase was to use electroencephalographic (EEG) recordings to measure the latency of the N20 component of the somatosensory evoked potential (SEP) elicited by ulnar nerve stimulation. A tightly fitting cap (Electro-cap International Inc., Eaton, OH, USA) was placed on the subject’s head with the reference electrode on the left ear lobe and the ground on the right ear lobe. The EEG signal was amplified, bandpass filtered (0.1–100 Hz), sampled at 2000 Hz and fed into a Biopac MP-150 wireless unit. Three hundred stimuli were delivered to the ulnar nerve with random interstimulus intervals of between 300 and 500 ms. The SEP at the C3 electrode was obtained by averaging the signal from all 300 stimulations and the latency of the N20 component was measured. At the end of this step the EEG cap was removed. Stimulus delivery and EEG data acquisition were controlled by Acknowledge 4.1 Software (Biopac System, Goleta, CA, USA).

The third and final step in the preparation phase consisted of finding the optimal stimulation site and intensity to provoke a response in the right first dorsal interosseous (FDI) with single pulse transcranial magnetic stimulation (TMS). Electromyographic (EMG) activity was recorded from the right FDI using Ag/AgCl electrodes placed in a belly-tendon montage. The EMG signal was amplified, bandpass filtered (20–1000 Hz) and digitised at a sampling rate of 2 kHz. TMS pulses were delivered with a Magstim BiStim^2^ stimulator (used in single High pulse mode) connected to a 70 mm figure-of-eight coil. The coil was placed over the left motor cortex with the handle pointing backwards at an angle of approximately 45° relative to the sagittal plane. The scalp position at which motor potentials (MEP) in the right FDI were evoked with the lowest stimulation intensity was identified as the FDI hotspot and all stimuli were delivered at this site in the Experimental Phase. A frameless stereotaxic neuronavigation system (Brainsight, Rogue Research, Montreal, QC, Canada) was used to maintain reliable coil positioning throughout the experiment. The resting motor threshold was measured at the hotspot as well as the stimulation intensity that evoked MEPs of approximately 1 mV (peak-to-peak amplitude) in the fully relaxed FDI. Throughout the Preparation and Experimental phases background FDI EMG was carefully monitored to ensure complete muscle relaxation. In addition, biceps brachialis EMG was recorded throughout the experiment to ensure that the arm remained completely at rest (i.e., that there were no withdrawal responses during painful stimuli).

#### 2.2.2. Experimental Phase

SAI is tested by delivering a peripheral electrical stimulus prior to a TMS pulse over the motor cortex. During the experimental phase SAI was tested either in the absence of pain (NEUTRAL) or when a thermal stimulation produced transient heat pain (PAIN). Four different interstimulus intervals (ISIs) between the peripheral stimulus and TMS pulse were tested: N20−5 ms, N20+2 ms, N20+4 ms and N20+10 ms (MEP_cond_) plus a condition in which TMS was applied alone (MEP_test_). Since MEP inhibition is maximal shortly after the occurrence of the N20 [17,20,33] we expected to see significantly smaller MEPs (relative to MEP_test_) at N20+2 and N20+4 but not at N20-5 or N20+10. Each condition (NEUTRAL/PAIN) was tested in a separate block and each block was repeated twice with the order of presentation counter-balanced across subjects. Each block consisted of 10 MEP_test_ trials and 20 MEP_cond_ trials (five per ISI), making a total of 60 trials per condition.

Throughout the experimental phase a 27 mm diameter thermode (CHEPS, Medoc Ltd, Ramat-Yoshai, Israel) embedded in the arm support was applied to the side of the hand (over the abductor digiti minimi muscle belly). The baseline temperature was set at 32 °C and during PAIN trials the temperature was increased to 50 °C by rapidly increasing the temperature (ramp-up time of approximately 500 ms) and maintaining the temperature at 50 °C for 800 ms. The onset of the temperature increase was triggered 750 ms before the electrical stimulus (for MEP_cond_ trials) or before the magnetic pulse (for MEP_test_ trials) in order to ensure that all stimulations occurred during the 800 ms plateau when the temperature was maintained at 50 °C (see Figure 1). After each PAIN block (30 trials/block), subjects were asked to rate the average pain intensity they felt across the entire block using an 11-point numerical rating scale (0–10) on which 0 represented no pain and 10 the worst pain they could imagine.

All stimulation devices (thermode, electrical stimulator, TMS) were triggered using Spike2 and a CED 1401 interface (Cambridge Electronic Design, Cambridge, UK) which also recorded motor evoked potentials from the right FDI.

### 2.3. Data Reduction and Statistical Analysis

The peak-to-peak amplitude of all MEPs was measured using a custom-made program (Spike2). For each condition, data from the two blocks were pooled and analysed using a two-way repeated measures ANOVA with factors Condition (NEUTRAL, PAIN) and Stimulation Interval (MEP_test_, N20−5, N20+2, N20+4, N20+10). Planned comparisons (each ISI versus MEP_test_) with Sidak adjustment for multiple comparisons were used to perform post-hoc tests. For each individual the percentage of SAI for each of the four ISIs was calculated as follows: [(MEP_cond_ − MEP_test_)/(MEP_test_)] × 100. Therefore positive values reflect facilitation and negative values reflects inhibition related to the presence of the conditioning stimulus. Means are presented with standard deviations in parentheses and Pearson correlations were used to explore relationships between variables. All statistical analyses were computed using SPSS 13.0 (SPSS Inc. Chicago, IL, USA) for Windows.

## 3. Results

### 3.1. Stimulation Parameters

The average stimulation intensity to evoke an M-wave amplitude of between 5% and 10% of the M-Max was 12.5 (2.6) mA. The mean latency of the N20 evoked by electrical stimulation of the ulnar nerve was 20.5 (1.0) ms. The average TMS intensity that evoked a MEP of approximately 1 mV in the FDI was 42.7% (8.3) of the maximal stimulator output. This corresponded to 122 (11) % of the resting motor threshold. The thermal stimulation was reported as painful by all subjects, and the mean pain intensity across the two PAIN blocks was 3.7/10 (2.0) on the numerical rating scale.

### 3.2. Effect of Pain on Short Afferent Inhibition

**Figure 2:**
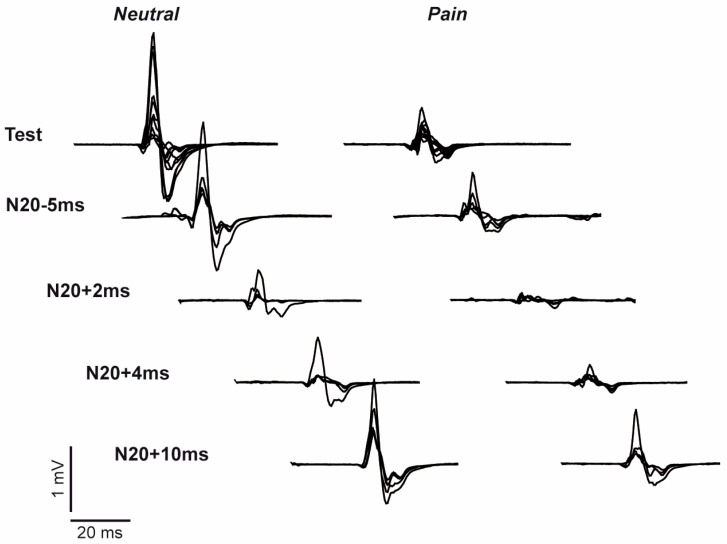
Example MEPs. This figure shows all 30 MEPs (10 tests and five for each ISI) from a single block of the NEUTRAL (**left panel**) and PAIN (**right panel**) conditions from a representative subject.

**Figure 3:**
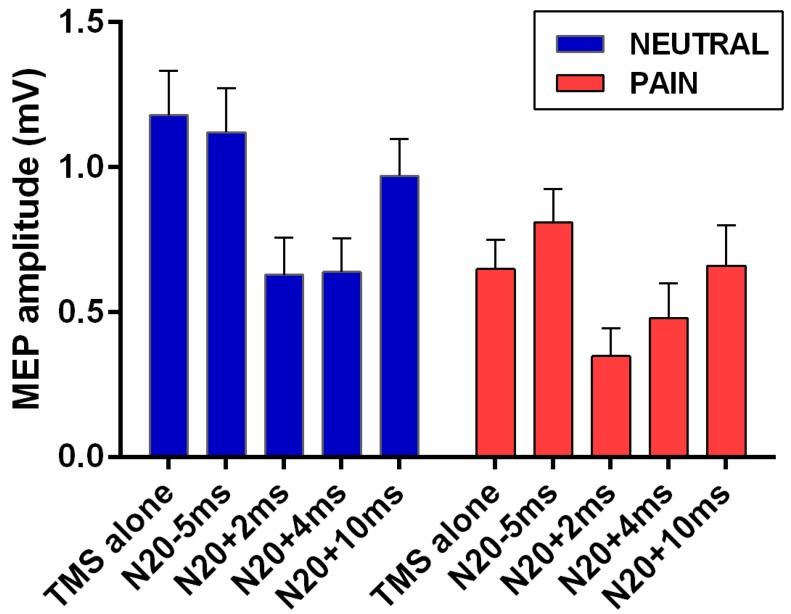
MEP amplitude as a function of Condition and Stimulation Interval. The average MEP amplitude for TMS alone and each of the four ISIs is shown separately for the NEUTRAL and PAIN conditions. Error bars indicate the standard error of the mean.

Figure 2 provides an example of raw MEPs evoked by TMS in the absence of peripheral stimulation (MEP_test_) and at each of the four ISIs (MEP_cond_) between the peripheral stimulus and the TMS pulse. Visual inspection of this figure shows that MEPs were smaller in the PAIN than in the NEUTRAL condition, and that for both conditions MEP amplitudes were inhibited (relative to MEP_test_) at the N20+2 and N20+4 ISIs. MEP amplitudes averaged across all blocks and all subjects (Figure 3) showed a similar pattern. The rmANOVA performed on the data in Figure 3 revealed that MEPs were significantly smaller in the PAIN condition (main effect of Condition: *p* = 0.005; average change = −37.4 (38.8) %) and that they varied across Stimulation Intervals (main effect of Stimulation Interval: *p* < 0.001). Post-hoc analyses showed that MEPs were significantly reduced compared to MEP_test_ at two ISIs: N20+2 ms (*p* < 0.001) and N20+4 ms, (*p* = 0.002). There was no interaction between Condition and Stimulation Interval (*p* = 0.189), indicating that PAIN did not affect SAI.

**Figure 4:**
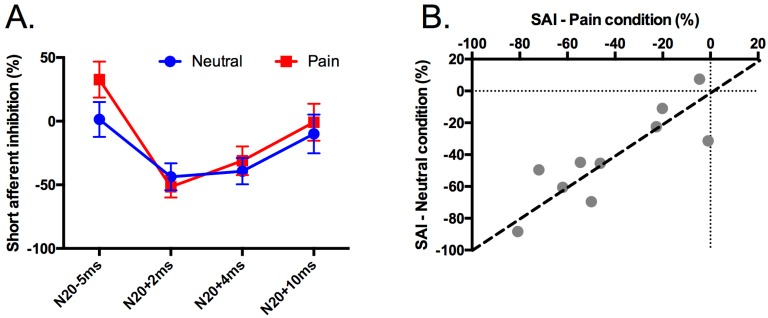
Percentage change in MEP amplitude. (**A**) The amount of SAI (percentage change in MEP amplitude) separately for each ISI for the NEUTRAL (Blue) and PAIN (Red) conditions. (**B**) The average amount of SAI at N20+2 and N20+4 in the NEUTRAL condition versus the PAIN condition. These two ISIs were averaged because they were significantly different from MEP_test_ (see [20] for a similar approach).

The left panel of Figure 4 shows the percentage of change in the MEP_cond_ amplitude relative to MEPtest separately for each ISI and each Condition. The substantial overlap of the two curves illustrates not only the absence of an interaction, but also that the percentage change in the MEP amplitude was similar in both conditions. To investigate whether this effect was present only in the averaged data, or whether the amount of inhibition exhibited by each subject was indeed very similar in both conditions, we plotted the percentage of inhibition averaged across N20+2 and N20+4 (the two ISIs that were significantly different from MEP_test_) in the NEUTRAL condition (average SAI = −41.6% (28.4)) against the same average measured during the PAIN condition (average SAI = −41.4% (27.8)) (right panel of Figure 4). This figure shows that almost all subjects showed inhibition in both conditions. In addition, there was a significant correlation (*p* = 0.002) between the amount of SAI in each condition, with SAI in the NEUTRAL condition explaining 71.6% of the variance in SAI in the PAIN condition. We interpret this as evidence that the absence of a difference in SAI between the NEUTRAL and PAIN conditions cannot simply be explained by high intra-subject variability in SAI. Rather, the amount of inhibition for a given subject was very similar in both conditions (all points in Figure 4 fall close to the identity line), suggesting that the amount of SAI within each individual is very stable.

Finally, we explored whether the percentage of inhibition of MEP_test_ in the PAIN condition relative to the NEUTRAL condition was associated with pain intensity. In agreement with previous findings obtained with the same experimental paradigm [21], no significant relationship was found (*p* = 0.488).

## 4. Discussion

In this study we investigated whether heat pain applied to the hand interferes with sensorimotor integration of a hand muscle as measured with SAI. The results showed (1) that muscle responses were inhibited in the presence of pain; (2) that significant inhibition was observed when the TMS pulse occurred 2 or 4 ms after the arrival of the afferent volley in S1; and (3) that the percentage of SAI was similar in both the presence and absence of heat pain.

The observation that heat pain inhibited FDI motor responses by approximately 40% is consistent with several other studies showing that experimental cutaneous and muscle hand pain reduce the corticospinal excitability of hand muscles [21,22,23,24,25,26,27,28,29]. Since smaller MEP_test_ amplitudes were expected in the PAIN condition, we could have increased the stimulation intensity to ensure comparable MEP_test_ amplitudes in the NEUTRAL and PAIN conditions. Instead we chose to keep the stimulation intensity constant across conditions because previous studies showed that the amount of SAI in FDI is robust for MEP_test_ amplitudes between 0.2 and 1 mV [34,35]. Although this choice raises the possibility that a different (smaller) population of intracortical interneurons was being stimulated, increasing the intensity of the stimulation would bear an opposite risk. This choice was also motivated by the observation that inhibition provoked by paired-pulse TMS over the motor cortex is abolished when MEP_test_ is above 130% of the motor threshold [36]. It is important to note that the reduced MEP_test_ amplitude during pain did not result in a floor effect (i.e., the impossibility of observing inhibition) which could have limited our ability to observed pain-related modulation in SAI, as MEP_test_ amplitudes of 1 mV and 0.5 mV (amplitudes close to what we observed in our NEUTRAL and PAIN conditions) result in similar amounts of SAI [37]. This idea is further supported by the fact that the amount of SAI in the NEUTRAL and PAIN conditions was highly correlated and similar to the amount reported in previous studies (e.g., [20]).

Despite the fact that acute pain has been shown to alter sensory processing and reduce corticospinal output [21,22,23,24,25,26,27,28,29,30,31], the present study suggests that it does not interfere with sensorimotor integration as tested with SAI using short pulses of cutaneous heat pain. The lack of change in SAI during exposure to acute pain is in accordance with the only previous study of SAI and pain that used a muscle pain model [28]. It is also consistent with the results of a recent study which reported similar temporal profiles for changes in both S1 and M1 in response to muscle pain, as well as an association between changes in both areas across subjects during pain [29]. Indeed, if pain-induced changes in S1 and M1 are co-modulated both in time and amplitude, they are unlikely to lead to dysfunctional sensorimotor integration. Interestingly, however, since after the resolution of pain S1 and M1 changes are still present but no longer correlated, alterations in sensorimotor integration might be more likely after pain resolution rather than during pain. This fits with the results of another study from the same group using the same pain model showing altered SAI and long-latency afferent inhibition (LAI) after the resolution of pain [28]. The reason why sensorimotor integration would be affected only after the resolution of pain, but not during, is unclear at this stage. While it could be a response to pain resolution, an alternative possibility is that these changes reflect a delayed response to pain exposure. Performing studies with pain exposure of variable durations across groups or longer pain durations (for example the capsaicin model for cutaneous pain [22], the nerve growth factor (NGF) injection model for muscle pain [38], or a laser-paired-associative stimulation paradigm [39]) would help clarify the time course of changes in SAI, and whether changes in SAI play any role in the sensorimotor disturbances observed in acute or chronic pain.

One limitation of the TMS technique used in the present study is that it cannot discriminate whether the reduction in MEP amplitude observed in the presence of pain was due to cortical or spinal mechanisms. However, the results of previous studies using complementary techniques such as transcranial electrical stimulation or H-reflex testing suggest that at least part of these effects can be attributed to a modulation of motor cortex excitability [22,24,25,26,27]. Another limitation is that the effect of pain on the excitability of the somatosensory cortex was not assessed. It would be of interest in further studies to assess both pain-induced modulation in S1 excitability and SAI given the results of recent studies showing that a decrease in S1 excitability induced by non-invasive stimulation results in a reduction of the amount of SAI. At this stage, the impact of nociceptive stimuli on S1 excitability (as measured by the short latency components of somatosensory evoked potentials) remains unclear, especially for cutaneous nociceptive modalities [30,31,40,41,42,43,44,45]. One also needs to consider that the nociceptive modality and the location of the nociceptive stimuli with respect to the tested muscle might influence the results. In the present study, the skin region where the thermode was applied was remote from the tested muscle (although both are innervated by the ulnar nerve, the nerve that was stimulated to induce SAI). A potential impact of the pain modality is supported by studies looking at the influence of pain on other intracortical inhibitory networks in M1. During cutaneous pain, a decrease in short-interval intracortical inhibition (SICI) has been observed [23], while during muscle or joint pain SICI was reported to be unchanged [46,47,48] (but increased after pain resolution [48]).

The lack of evidence that either experimental cutaneous (present study) or muscle pain [28] affects SAI, together with the relatively limited evidence of disrupted SAI in patient populations [18], leave open the question of the mechanisms underlying the sensorimotor integration disturbances that are observed in individuals with pain. As pointed out in the introduction, sensory inputs influence motor cortex activity through several pathways, and only the most direct pathways are assessed using SAI. Thus, interactions between pain and sensorimotor integration disturbances might involve higher-order integrative areas. The results of a recent study comparing EEG cortical sources in healthy subjects under conditions of sensorimotor congruence or incongruence suggest that this might be the case [49]. In this study, the authors reported that sensorimotor incongruence was associated with increased activation in the right posterior parietal cortex (PPC). Interestingly, when subjects were classified according to the amount of discomfort generated during sensorimotor incongruence, individuals who were highly sensitive to discomfort exhibited more activation in two key pain-related areas: the anterior cingulate cortex (ACC) and posterior cingulate cortex (PCC). Since the incongruent condition produced discomfort only and not pain, the use of this type of paradigm in individuals who are highly susceptible to feelings of pain in response to sensorimotor incongruence, such as individuals with chronic pain, could help determine whether ACC and PCC activations can be directly related to pain modulation. This approach could also help clarify whether pain contributes to increased sensitivity to sensorimotor incongruence by comparing PPC modulation between healthy individuals and chronic pain patients. Moreover, the interplay between PPC and M1 could be probed using TMS, a method that has already been used to show that parietal-motor interactions are modulated during sensorimotor training [50], but which has never been applied to investigations of sensorimotor integration in the context of pain.

## 5. Conclusions

In conclusion, the present study showed that while cutaneous heat pain of short duration applied to the hand can cause a marked decrease in the corticospinal excitability of a hand muscle, it does not affect SAI. It remains possible that longer pain durations might affect SAI, but at this point the evidence for altered SAI under conditions of acute or chronic pain remains limited. More studies are needed to assess whether other parietal-motor pathways are involved in the interactions between sensorimotor integration and pain that are observed in behavioural and clinical studies.
